# Phosphatidylglycerol to Treat Chronic Skin Wounds in Diabetes

**DOI:** 10.3390/pharmaceutics15051497

**Published:** 2023-05-14

**Authors:** Yonghong Luo, Edymarie Vivaldi Marrero, Vivek Choudhary, Wendy B. Bollag

**Affiliations:** 1 Department of Physiology, Medical College of Georgia at Augusta University, Augusta, GA 30912, USA; yoluo@augusta.edu (Y.L.); 121evivaldi@uccaribe.edu (E.V.M.); vchoudhary@augusta.edu (V.C.); 2 Charlie Norwood VA Medical Center, One Freedom Way, Augusta, GA 30904, USA; 3 Department of Dermatology, Medical College of Georgia at Augusta University, Augusta, GA 30912, USA

**Keywords:** aquaporin-3 (AQP3), chronic wounds, diabetes, dioleoylphosphatidylglycerol (DOPG), inflammation, mitochondrial function, phosphatidylglycerol, phospholipase D2 (PLD2), wound healing

## Abstract

This review proposes the use of dioleoylphosphatidylglycerol (DOPG) to enhance diabetic wound healing. Initially, the characteristics of diabetic wounds are examined, focusing on the epidermis. Hyperglycemia accompanying diabetes results in enhanced inflammation and oxidative stress in part through the generation of advanced glycation end-products (AGEs), in which glucose is conjugated to macromolecules. These AGEs activate inflammatory pathways; oxidative stress results from increased reactive oxygen species generation by mitochondria rendered dysfunctional by hyperglycemia. These factors work together to reduce the ability of keratinocytes to restore epidermal integrity, contributing to chronic diabetic wounds. DOPG has a pro-proliferative action on keratinocytes (through an unclear mechanism) and exerts an anti-inflammatory effect on keratinocytes and the innate immune system by inhibiting the activation of Toll-like receptors. DOPG has also been found to enhance macrophage mitochondrial function. Since these DOPG effects would be expected to counteract the increased oxidative stress (attributable in part to mitochondrial dysfunction), decreased keratinocyte proliferation, and enhanced inflammation that characterize chronic diabetic wounds, DOPG may be useful in stimulating wound healing. To date, efficacious therapies to promote the healing of chronic diabetic wounds are largely lacking; thus, DOPG may be added to the armamentarium of drugs to enhance diabetic wound healing.

## 1. Introduction

Diabetes mellitus afflicts more than 34 million Americans, or about 13% of the adult population in the United States. In 2017, treatment was estimated to cost $327 billion [1]. Diabetes predisposes affected individuals to a variety of co-morbidities, including cardiovascular disease, kidney disease, and vision problems [1]. Another important co-morbidity associated with diabetes is impaired skin wound healing [2]. Impaired skin wound healing is also observed in aged skin, and this deficiency can lead to the development of non-healing (chronic) wounds [3]. Many abnormalities have been found in non-healing or chronic wounds, including compromised re-epithelialization, increased inflammation and oxidative stress, decreased nerve density, and impaired angiogenesis [4,5]. Low-grade infection can also contribute to the chronicity of these wounds [4,5]; in turn, non-healing wounds are a major medical and financial burden on the healthcare system of the United States, affecting approximately 6.5 million patients and costing a reported $25 billion or more annually in treatment expenses [6]. In patients with diabetes, in particular, non-healing wounds of the lower extremities result in more than 125,000 amputations annually [1]. However, the pathological mechanisms underlying this condition are unclear, and effective treatments are largely lacking. Thus, therapies to accelerate the impaired healing of chronic skin wounds are critically needed.

Diabetes, in particular type 2 diabetes mellitus (T2DM), is characterized by major metabolic changes including insulin resistance, hyperglycemia, oxidative stress, and an inflammatory state. Physiologically, insulin induces the recruitment of glucose transporters to the plasma membrane of muscle cells and adipocytes, among other cell types, to allow uptake of glucose from the blood into these cells. Insulin resistance impairs this signaling pathway and results in hyperglycemia as these normally insulin-sensitive tissues instead use fatty acids as a fuel source [7]. The oxidative stress associated with T2DM occurs when endogenous antioxidant systems are overwhelmed by the excess reactive oxygen species (ROS); however, these mechanisms are incompletely understood [8], but likely involve hyperglycemia and enhanced production of ROS as a result of deleterious changes in mitochondrial function. Metabolites called advanced glycation end-products (AGEs), formed as a result of hyperglycemia and oxidative stress (please see the next section), can induce inflammation through interaction with the receptor for AGEs (RAGE), which activates the pro-inflammatory transcription factor, the nuclear factor kappa-light chain-enhancer of activated B cells (NFκB). Together these factors, AGEs, oxidative stress, and inflammation, are thought to act to inhibit wound healing in individuals with diabetes. This review discusses each of these elements and their effects on the predominant cells of the skin epidermis, the keratinocytes, as well as a possible therapeutic option, the anionic phospholipid dioleoylphosphatidylglycerol (DOPG), which can inhibit many of these effects. Based on the known actions of DOPG on inflammation [9,10,11,12,13,14,15,16,17,18], as well as limited information indicating its ability to improve mitochondrial function [19] and thus oxidative stress, we propose DOPG as a potentially safe and effective treatment to enhance diabetic wound healing. The goal of this review is to discuss the evidence supporting this idea.

## 2. Mitochondria, ROS Generation, and Formation of AGEs in Diabetes

### 2.1. ROS Generation

**Figure 1:**
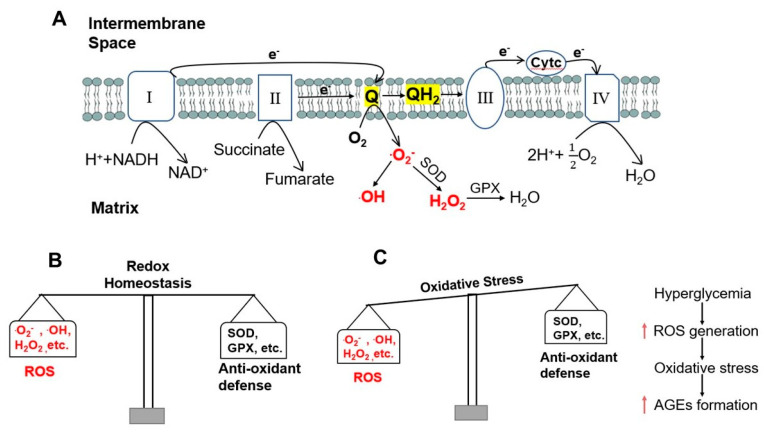
ROS generation and oxidative stress induced by hyperglycemia. (**A**) Electrons pass through the four complexes of the respiratory chain and ROS formation in mitochondria. Electrons from complexes I and II are transferred to ubiquinone (Q) and Q is reduced to ubiquinol (QH_2_). QH_2_ passes electrons to complex III, which passes them to a small mobile protein, cytochrome c (Cytc), and then to complex IV. When the entry of electrons and/or the flow of electrons through the respiratory chain are imbalanced, the intermediate radical ∙Q^−^ can donate an electron to oxygen (O_2_) to produce superoxide free radical ∙O_2_^−^. ∙O_2_^−^ can form hydroxyl free radical ∙OH or can be converted to hydrogen peroxide (H_2_O_2_) by superoxide dismutase (SOD). H_2_O_2_ is further converted to water (H_2_O) by glutathione peroxidase (GPX). (**B**) Redox homeostasis under normal conditions. (**C**) Oxidative stress in hyperglycemia increases the formation of AGEs.

Oxidative phosphorylation is an effective energy-yielding mechanism in aerobic organisms [20]. During oxidative phosphorylation, electrons from NADH pass through the respiratory chain complex I to ubiquinone Q, or electrons from succinate pass through complex II to ubiquinone Q; electrons from glycerol 3-phosphate and from β-oxidation of fatty acids also pass to ubiquinone Q. Ubiquinone Q is thus reduced to ubiquinol QH_2_, which acts as a mobile carrier of electrons and protons. Electrons are passed from QH_2_ to complex III and then to complex IV, where electrons are donated to oxygen (O_2_) to reduce O_2_ to water (H_2_O). When the input of electrons and/or the transfer of electrons through the respiratory chain are imbalanced, an electron can be passed from the intermediate radical ^.^Q^−^ to O_2_ to form superoxide free radical ^.^O_2_^−^. This highly reactive superoxide free radical can form the even more reactive hydroxyl free radical, ^.^OH [21]. Accumulation of these ROS results in damage to DNA, RNA, proteins, and ultimately cells, tissues, and organs. To protect cells from oxidative damage, superoxide dismutase converts the superoxide free radical ^.^O_2_^−^ to hydrogen peroxide, H_2_O_2_, which is further converted to H_2_O by glutathione peroxidase [22] (Figure 1A). However, these and other endogenous anti-oxidant mechanisms can be overwhelmed by excessive ROS generation to result in a state of oxidative stress (Figure 1B,C).

### 2.2. The Role of Mitochondrial ROS and Hyperglycemia in the Formation of AGEs

As indicated above, along with hyperglycemia, oxidative stress is another major characteristic metabolic change in T2DM. Oxidative stress can be induced by hyperglycemia, as shown by Nishikawa et al. [23]. In their experiment, Nishikawa and colleagues showed that hyperglycemia induced the formation of advanced glycation end products (AGEs) in bovine aortic endothelial cells. However, when these authors incubated these cells with thenoyltrifluoroacetone (TTFA, an inhibitor of complex II), carbonyl cyanide m-chlorophenylhydrazone (CCCP, an uncoupler of oxidative phosphorylation), uncoupling protein-1 (UCP1, a specific protein uncoupler of oxidative phosphorylation), or manganese superoxide dismutase (Mn-SOD), they found that AGEs formation induced by hyperglycemia was prevented [23]. These data indicate that movement of electrons through the electron transport chain and the production of ROS when the electrons “leak” is a causal link between hyperglycemia and AGE formation (Figure 1C).

### 2.3. Formation of AGEs

**Figure 2:**
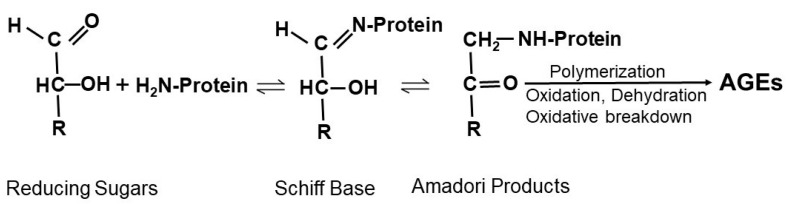
Schematic showing the Maillard reaction. Nucleophilic addition of free amino groups of proteins to carbonyl groups of reducing sugars leads to the formation of a Schiff base. Subsequent rearrangement of the Schiff base generates Amadori products. Amadori products undergo irreversible biochemical reactions including polymerization, oxidation, dehydration, and oxidative breakdown to form various AGEs.

AGEs are formed through non-enzymatic reactions between reducing sugars and proteins, lipids, or nucleic acids under hyperglycemic and oxidative stress states. As shown below in Figure 2, the non-enzymatic reaction between reducing sugar and free amino groups of amino acids form a reversible and non-stable Schiff base, which is further rearranged to form a more stable but also reversible Amadori product. Amadori products can undergo various reactions involving oxidation, dehydration, polymerization, and oxidative breakdown to form various AGEs [24,25,26]. Reducing sugars and free amino groups on DNA can undergo Amadori reactions to lead to the formation of DNA-AGE [27]. Lipid peroxidation can also generate advanced lipoxidation end-products [28]. Thus, AGEs form from non-enzymatic glycation of proteins and other macromolecules during the hyperglycemia in diabetes [29], particularly under conditions of increased oxidative stress. 

There is growing evidence that AGEs may play a role in insulin resistance in T2DM. Tan et al. [30] and Tahara et al. [31] independently conducted clinical studies of 207 (97 males, 110 females) non-obese, non-diabetic subjects and 322 (216 males, 106 females) non-diabetic Japanese subjects, respectively. They found that serum levels of AGEs were positively associated with insulin resistance in these nondiabetic subjects. Indeed, Pinto-Junior et al. [32] showed that AGE-albumin induced insulin resistance in healthy rats and the mechanism likely related to the ability of AGEs to down-regulate GLUT4 (encoded by the gene Slc2a4) in skeletal muscle. In this study, the authors treated healthy rats with AGE-albumin for 12 weeks and found that the body mass and blood glucose, and plasma insulin concentrations of the rats were unchanged. However, the rate of blood glucose decay during insulin tolerance tests was decreased [32]. Slc2a4/GLUT4 expression, both in response to AGE-albumin treatment in vivo and to the incubation of rat skeletal muscle with AGE-albumin in vitro (for 5 and 7 h), was reduced compared to the control, suggesting that AGEs repress GLUT4 expression to induce insulin resistance. AGEs are known to bind to and activate the receptor for AGEs or RAGE (encoded for by the gene AGER), which is expressed in a variety of tissues, including the skin. Since diabetes induces skin dysfunction, with both impaired wound healing and skin dryness (xerosis) common in patients with the disease [33], it seems possible that AGEs could potentially mediate, at least in part, the adverse skin effects of diabetes, contributing to the morbidity associated with this disease.

## 3. Skin Structure and Function

**Figure 3:**
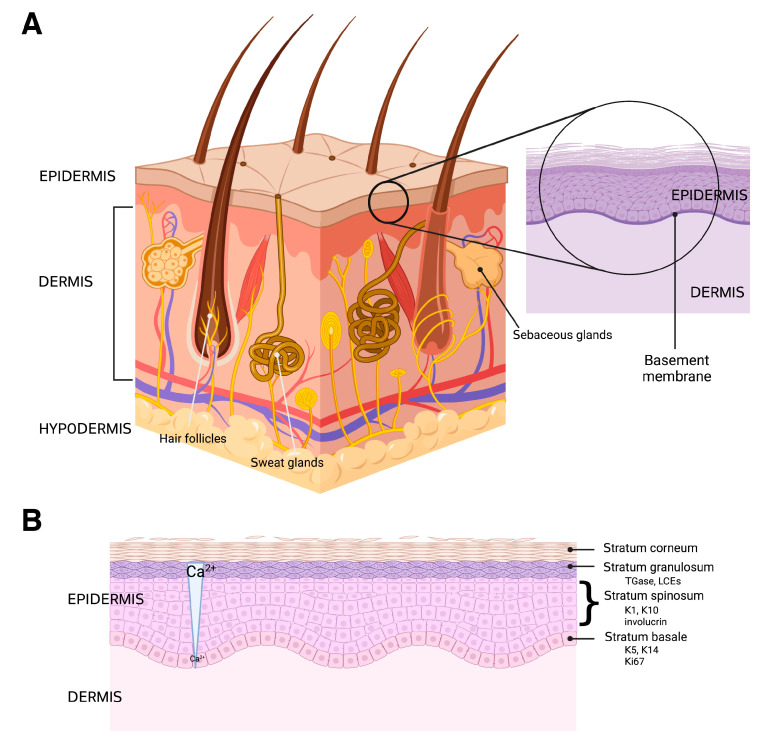
The structure of the skin and epidermis is shown. (**A**) The skin is composed of three layers: the epidermis, dermis, and hypodermis, as well as numerous skin appendages, such as hair follicles, sweat glands, and sebaceous glands. The dermis is vascularized and enervated as well, providing touch sensation, thermodetection, and other functions. (**B**) The epidermis has multiple layers (strata) characterized by specific markers, including the following: keratin (K)-5 and K14 and Ki67 (a proliferative marker) in the basal layer (stratum basale); K1, K10 and involucrin in the spinous layer (stratum spinosum); and transglutaminase (TGase), which crosslinks proteins such as the late cornified envelope proteins (LCEs) to form a cornified shell underneath the plasma membrane in the granular layer (stratum granulosum). The cornified layer (stratum corneum) consists of squames within a lipid matrix. The differentiation process is thought to be regulated, at least in part, by a calcium concentration gradient, with low levels in the basal layer and gradually increasing calcium concentrations into the granular layer. Created with Biorender.com.

The skin is the major protective barrier of the human body and the site of many dynamic processes including temperature homeostasis, synthesis of melanin, and sensation. It is composed of three distinct layers: the epidermis, dermis, and hypodermis, all of which vary significantly in their anatomy and function [34] (Figure 3A). 

### 3.1. The Hypodermis and Dermis

The hypodermis, also called subcutaneous tissue, is the deepest layer of the skin and its width varies within the body, depending on the region. It is mainly composed of adipose tissue, which works as an insulator. The abundant triglyceride within the adipose tissue also accounts for the energy storage function of the hypodermis [35]. 

The dermis is the intermediate and thickest layer of the skin, consisting predominantly of connective tissue. The dermis is mainly composed of collagen type I and III and elastic fibers [36,37], with its structure providing strength, support, protection, and flexibility to the skin and deeper structures [36]. It is divided into two layers: papillary and reticular dermis, which are characterized by their locations (more superficial versus deeper, respectively) and morphology (highly vascularized loose versus dense connective tissue, respectively). The dermis also contains hair follicles and blood vessels. The blood vessels that compose the dermal vasculature of the dermis are made up of the superficial and lower intercommunicating plexuses [37], which are essential to the process of thermoregulation. Along with the heat exchange regulated by contraction and dilatation of blood vessels, the dermis also houses sweat glands, which can additionally contribute to thermoregulation through evaporative heat loss. Sweat glands are appendages of the skin and can be categorized as eccrine, apocrine, or apoeccrine [38,39]. Eccrine glands are the most abundant type, distributed essentially throughout the body [39], and are the sweat glands responsible for thermoregulation, whereas the apocrine glands are specialized scent glands in the skin. It has been stated that eccrine sweat has an important role in epidermal barrier homeostasis (described below), as it transports water and antimicrobial peptides to the skin surface [39,40]. Through the constituents of sweat, such as lactate, urea, sodium, and potassium, the eccrine glands help to maintain physiologic skin hydration [41]. In addition, these eccrine glands also have an immune function, as Rieg et al. [41] have shown that sweating leads to a decrease in the number of viable bacteria on the surface of the skin. Finally, the dermis houses nerve endings, which are responsible for sensations such as pain, pressure, and itchiness. The dermis and the epidermis, the latter of which is the outermost layer of the skin, are joined by the dermal–epidermal junction (DEJ). The DEJ is composed of basal keratinocyte products and structural proteins, such as plectin isoform 1a, BP230, BP180, α6β4 integrin, and tetraspanin CD151 [42]. The main function of this basement membrane is to protect against mechanical shear; however, it also plays a role in signal transduction and cell differentiation. 

### 3.2. The Epidermis

The most superficial layer of the skin is the epidermis, which functions as a barrier to protect the body from the environment, including microbes, physical trauma, and chemical insults. It is arranged in four layers, from the most superficial portion to the deepest: the stratum corneum, the stratum granulosum, the stratum spinosum, and the stratum basale. The epidermis is composed of a variety of cell types, including keratinocytes, melanocytes, Langerhans cells, and Merkel cells [6], although keratinocytes constitute approximately 90% of the tissue. Merkel cells are specialized cells that exhibit a neuroendocrine function due to their closeness to nerve endings, as they receive the sensation of touch, producing substances that act as hormones [43]. Langerhans cells are specialized dendritic cells responsible for initiating certain immune responses [44,45]. Melanocytes produce melanin, which is a pigment that protects the body from ultraviolet (UV) radiation that may damage DNA, and thus lead to skin cancer. Keratinocytes, as previously mentioned, are the most dominant cell type of the epidermis [46] and create the epidermal barrier as they differentiate from the basal layer to the stratum corneum. The process of keratinocyte differentiation is thought to be regulated by a calcium gradient within the epidermis (Figure 3B). The differentiation process also involves reprogramming of gene expression and cell morphology [47], with the cells expressing different markers as they progress through the various epidermal layers (Figure 3B). The main purpose of these cells is to form a barrier that protects against microbial, viral, fungal, and parasitic invasion, as well as UV radiation, and to minimize heat, solute, and water loss [48]. If a disruption occurs in the differentiation process the epidermal barrier’s protective functions may decline. Diseases such as psoriasis, atopic dermatitis, and allergic contact dermatitis are likely related to disruption in the keratinocyte differentiation process [49,50]. The proliferation and differentiation processes of keratinocytes are also of substantial importance in wound healing, with keratinocytes involved in the initiation, maintenance, and completion of this action [51].

Keratinocytes produce the intermediate filament proteins, keratins. In humans, there are 54 different types of keratins; specifically, keratinocytes in the basal layer express keratins 5 and 14, while the more differentiated spinous cells express keratins 1 and 10 [52]. Keratins are of great importance due to their many functions. They protect epithelial cells from mechanical damage that may result in cell death and also play a crucial role in processes such as transcriptional regulation, cell adhesion, migration, proliferation, angiogenesis, and inflammation [53]. In addition, keratin’s role in skin pigmentation has also become of great interest, as recent studies have demonstrated that this intermediate filament protein may be involved in pigmentary disorders [54,55]. The skin is the location of many diverse processes that are essential in maintaining the normal role of key components for the protection and maintenance of homeostasis in the body. Understanding the complexity and interrelation between all of skin’s components could be important for the prevention and treatment of diverse diseases, including the impaired wound healing (and xerosis) seen in diabetes.

### 3.3. Skin Wound Healing

Wounding of the skin is a problem for the organism, as it disrupts the epidermal barrier, potentially allowing the entry of microbes into the internal environment, with resultant infections possibly leading to disfigurement, disability, or even death. Therefore, the wound must be healed as rapidly as possible while also inhibiting microbial growth. Partial thickness wounds can heal by primary intention, with apposition of the wound edges via re-epithelialization (i.e., epithelial resurfacing) and wound contraction, whereas full thickness wounds require an initial formation of granulation tissue prior to re-epithelialization (reviewed in [56]). Wound healing typically exhibits three to four partially overlapping stages (reviewed in [57]). The initial stage involves hemostasis, or in other words, the formation of a blood clot to prevent continued bleeding, followed by inflammation, which is sometimes considered part of the hemostasis stage (reviewed in [58]). In the normal wound healing process, the inflammatory stage resolves to make way for the proliferative phase. Failure of the wound to transit from the inflammatory to the proliferative phase leads to impaired healing and can result in chronic wounds (reviewed in [46]). Subsequently, the remodeling stage is initiated in an attempt to restore the original tissue architecture [46]. Multiple cell types are involved in skin wound healing, but keratinocytes have been proposed to play an important role not only as structural cells but also as cells exerting immune functions during this process [46].

## 4. The Aquaporin 3-Phospholipase D2-Phosphatidylglycerol Signaling Pathway and Diabetes

### 4.1. Epidermal Aquaporin 3 (AQP3)

One protein that is highly expressed in epidermal keratinocytes and is important in many aspects of skin function, including wound healing (reviewed in [21]), is aquaporin-3 (AQP3). AQP3 is a channel that transports water, glycerol, and other small molecules such as hydrogen peroxide ([59,60] and reviewed in [61]). AQP3 is localized in essentially all of the layers of the epidermis and seems to play a role in many of the functions of this epithelium. Thus, numerous studies have shown the importance of AQP3 in skin (reviewed in [61,62]), with in vitro experiments underscoring an involvement of this protein in regulating keratinocyte proliferation, differentiation, and migration, and in vivo results pointing to its importance in the maintenance of skin moisture, barrier integrity, and skin wound healing [61,62]. Some of these AQP3 roles have been found based on studies of the AQP3 knockout mice created by Verkman and colleagues [63,64,65,66,67,68,69,70], which exhibit decreased hydration (conductance) and elasticity of the skin as well as delayed wound healing and permeability barrier repair after disruption [69]. Interestingly, the changes observed seem not to result from decreased epidermal water transport but rather from reduced glycerol content; in fact, the phenotype can be corrected by the administration (orally or topically) of pharmacologic amounts of glycerol, but not other osmotically active agents, such as propylene glycol, suggesting that glycerol is serving as more than just a simple moisturizing agent. In addition, these AQP3 knockout mice exhibit a resistance to epidermal hyperplasia/tumor formation in mouse models of skin carcinogenesis [67] and inflammatory skin diseases [71]. 

In vitro, these authors have further shown that proliferation and migration are reduced in keratinocytes derived from AQP3 knockout mice compared to wild-type mice [68]. In addition, AQP3 knockdown in human keratinocytes also inhibits cell proliferation and migration. On the other hand, co-expression of AQP3 with reporter genes, in which luciferase expression is driven by the promoters for keratin 1 or involucrin (markers of early and intermediate keratinocyte differentiation, respectively) results in increased luciferase activity under either basal conditions and/or differentiative conditions [72]. Furthermore, AQP3 re-expression in AQP3 knockout keratinocytes results in up-regulation of the expression of markers of keratinocyte differentiation, suggesting also a role for this channel in keratinocyte differentiation [73]. Therefore, accumulating evidence points to a crucial importance of AQP3 in the skin, an idea that is further supported by the data indicating its alteration in several skin diseases (reviewed in [61,62]).

### 4.2. AQP3 and Diabetes 

Data in the literature also suggest a role for AQP3 in the effect of diabetes on the skin. For example, AQP3 expression is reduced in the skin wounds of diabetic rats [74] and in aged skin [75,76,77], both conditions that are associated with impaired wound healing. Furthermore, data in the literature indicate that AQP3 expression is down-regulated in the unwounded epidermis of diabetic rodents [33,74,78]. The mechanism underlying this down-regulation was examined by Ikarashi et al. [33]. These authors demonstrated that one-week after the injection of streptozotocin (STZ), which destroys beta-cells in the islets of Langerhans to produce insulin deficiency, rodents exhibited hyperglycemia but normal epidermal AQP3 levels; however, two weeks after STZ administration, and despite similar blood glucose levels as at one-week post-STZ, AQP3 protein expression in the epidermis decreased [33]. This result suggests that some molecules, such as AGEs, which accumulate during diabetes, may underlie the reduction in AQP3 rather than the high blood glucose levels per se. Indeed, AGEs have been found to decrease AQP3 protein expression in mouse keratinocytes under certain conditions [79]. A role for AQP3 in the impaired wound healing of aged skin is also suggested by results showing that AQP3 levels are decreased in both extrinsically (sun-exposed) and intrinsically (chronologically) aged human skin [76,77] and in mouse skin [75]. Finally, in addition to effects on wound healing, AQP3 down-regulation has also been proposed to underlie the xeroderma (dry skin), which is often observed in individuals with diabetes [33,75]. Reduced AQP3 levels should lead to the decreased transport and epidermal content of glycerol, a natural humectant that holds water in the tissue; therefore, down-regulation of AQP3 in diabetic skin is consistent with the reduced skin hydration of diabetic xerosis.

### 4.3. AQP3 and the Generation of the Lipid Signal, Phosphatidylglycerol (PG)

**Figure 4:**
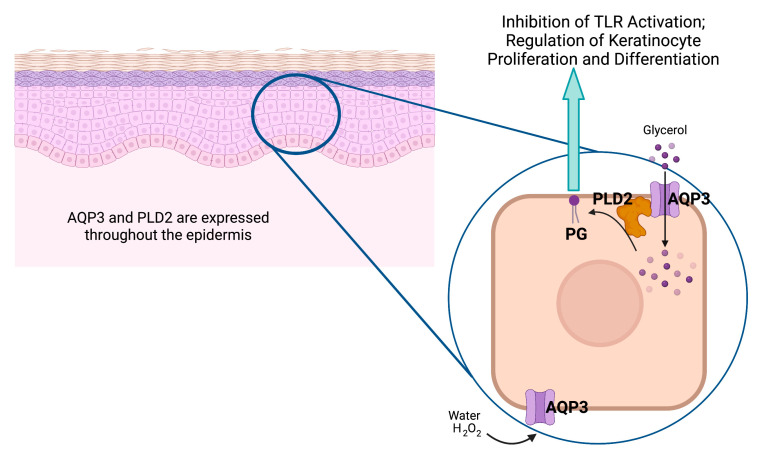
The aquaporin-3 (AQP3)/phospholipase-D2 (PLD2)/phosphatidylglycerol (PG) signaling pathway. Keratinocytes throughout the epidermis express AQP3, a water, glycerol, and hydrogen peroxide (H_2_O_2_) channel that associates with the lipid-metabolizing enzyme PLD2, also expressed throughout the epidermis [86]. PLD2 can convert the glycerol transported by AQP3 to the phospholipid PG, which serves as a signal to inhibit Toll-like receptor activation and regulate keratinocyte proliferation and differentiation (please see text). Created with Biorender.com (accessed 30 April 2023).

**Figure 5:**
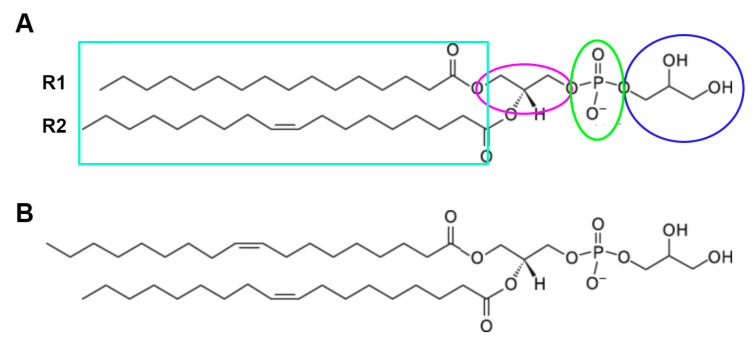
Phosphatidylglycerol. The structure of PG is shown, with the glycerol headgroup (circled in blue), the phosphate linker (green ellipse), and the glycerol backbone (magenta ellipse). The two fatty acid tails (R1 and R2 in the teal box) are also shown, each of which can be one of multiple fatty acids of varying carbon chain lengths and degrees of saturation. (**A**) The PG species illustrated is 1-palmitoyl-2-oleoyl-PG. (**B**) The structure of dioloeoylphosphatidylglycerol (DOPG) is shown. Adapted from the Avanti Lipids, Inc. website (https://avantilipids.com/product/841138; accessed on 30 April 2023).

The ability of AQP3 to transport glycerol also underlies another possible mechanism through which the channel may affect the skin—through the generation of the phospholipid signal phosphatidylglycerol (PG). We previously found that the phospholipase D (PLD) isoform, PLD2, colocalizes with AQP3 in epidermal keratinocytes [80]. In addition, we demonstrated that PLD2 is physically and functionally coupled to AQP3, with PLD2 able to convert AQP3-transported glycerol into PG [81] (Figure 4). In turn, PG serves as a novel lipid signal ([72] and reviewed in [61,62]) that regulates keratinocyte proliferation and differentiation in vitro [72] and accelerates skin wound healing in vivo [82]. Additionally, as discussed above, manipulation of the AQP3/PLD2 signaling pathway can promote keratinocyte differentiation, including by direct application of PG liposomes derived from egg [72]. Indeed, these egg PG liposomes can inhibit the proliferation of rapidly dividing keratinocytes and stimulate the growth of slowly proliferating cells [72], essentially normalizing keratinocyte function. Egg-derived PG represents a mixture of different PG species containing a variety of fatty acids at the R_1_ and R_2_ position of the glycerol backbone (see Table 1 and Figure 5), and we considered the possibility that different PG species, as defined by the various fatty acids attached to the lipid backbone, might induce different effects. In subsequent studies examining various PG species thought to comprise egg PG, we determined the species most efficacious at regulating keratinocyte proliferation versus differentiation, and the results can be summarized as follows: PG species comprising polyunsaturated fatty acids are most effective at inhibiting proliferation, whereas PG species containing monounsaturated and saturated fatty acids are best at promoting keratinocyte growth (Table 2) [83]. This ability of certain PGs to stimulate keratinocyte proliferation would be expected to enhance skin wound healing, whereas effects on differentiation might also be beneficial as complete epidermal regeneration requires both processes. Indeed, we previously demonstrated that egg PG (containing PGs with saturated, monounsaturated, and polyunsaturated fatty acids) accelerates the healing of a full-thickness skin wound [82]. However, these experiments also suggested that DOPG is more effective at stimulating keratinocyte proliferation (of slowly dividing cells) [83], and DOPG also enhances corneal epithelial wound healing, even in AQP3 knockout mice [84], which exhibit impaired corneal wound healing [85]. Therefore, we focused on this particular PG species as a potential treatment for skin wound healing, although other PG species, such as 1-palmitoyl-2-oleoylphosphatidylglycerol (POPG), might also show efficacy.

PG has recently been found to be altered in keratinocytes exposed to ultraviolet (UV) light and in certain skin diseases. Thus, after UV irradiation, liquid chromatography-mass spectrometry (LC-MS) analysis indicated that the levels of PG possessing fatty acids with a total of 36 carbons and 1 double bond (so potentially 1-stearoyl-2-oleoylphosphatidylglycerol, among other possibilities) were acutely elevated approximately three-fold [87]. After more chronic UV exposure, a PG-containing fatty acid with 36 carbons and no double bonds was elevated. PG was also increased in the plasma of subjects with chronic urticaria [87], as well as those with psoriasis [88]. In this latter study, the plasma PG was contained in exosomes, and it seems likely that differences in plasma lipids observed in individuals with skin disease arise from changes in lipids in extracellular vesicles derived from skin cells. Nevertheless, these reports provide no information as to whether PG serves as a cell signaling molecule and/or whether the alterations in PG play a role in the disease process. 

## 5. DOPG and Its Potential Beneficial Effects in Healing of Chronic Diabetic Wounds

### 5.1. DOPG and Inflammation

Chronic wounds are known to be characterized by inflammation [4,5]; in fact, these wounds seem to be “stuck” in the inflammatory stage [46]. In recent novel data, we have shown that, in skin, PG is anti-inflammatory such that topically applied soy PG, a mixture of PG species with a high proportion of polyunsaturated fatty acids, can inhibit the inflammation induced by a contact irritant in a mouse ear edema model [88]. However, in subsequent experiments investigating whether soy PG affects inflammatory mediator expression in response to innate immune system activation, soy PG was found to possess a narrow therapeutic window, with higher doses actually stimulating the expression of some cytokines [18]. Therefore, we focused instead on DOPG and demonstrated that this lipid inhibits the activation of pattern recognition receptors, in particular, Toll-like receptor-2 and -4 (TLR2 and TLR4) [15]. The activation of these pattern recognition receptors stimulates signaling through NFκB, with the resultant production and release of cytokines and chemokines, recruitment and activation of the innate immune system, inflammation and eventual involvement of the adaptive immune system. Therefore, DOPG’s ability to inhibit TLR2 and TLR4 in vitro should suppress inflammation in vivo.

In vitro, DOPG inhibits TLR2/4-induced cytokine production in response to microbial components, also known as pathogen-associated molecular patterns (PAMPs), as well as endogenous compounds released by threatened or damaged cells to alert the immune system to the presence of noxious stimuli—known as danger/damage-associated molecular patterns (DAMPs) [15]. DOPG suppresses both PAMP- and DAMP-induced inflammatory mediator production in primary mouse keratinocytes and a macrophage cell line [15]. The related PG species, POPG, has similarly been found to inhibit TLR2 and TLR4 activation in primary alveolar macrophages [12]. We also examined the effect of DOPG on inflammation in the imiquimod-induced mouse model of psoriasis and found that topical application of DOPG decreased the imiquimod-stimulated inflammation and TNFα immunoreactivity and improved skin lesions in this in vivo psoriasis model [15]. In addition, this anti-inflammatory effect occurs in the absence of a complete inhibition of the immune system, thereby likely circumventing the potential adverse side effects of many immune-targeted therapies (e.g., increased susceptibility to infection) [89]. Indeed, DOPG exerts minimal actions on the activation of TLR7 and TLR8, which recognize single-stranded RNA viruses and are targeted by imiquimod [15].

Other investigators have also observed the anti-inflammatory effects of PG. Thus, Voelker and colleagues demonstrated the ability of POPG found in pulmonary surfactant to inhibit the activation of TLR2 and TLR4 by microbial components (e.g., lipopolysaccharide (LPS)). These authors observed a similar but typically slightly less potent effect of another negatively charged phospholipid, phosphatidylinositiol. Similarly, Mäder and colleagues showed that DOPG-containing liposomes (30% DOPG + 70% soybean-derived phosphatidylcholine) reduced TNFα production by mouse peritoneal macrophages, with no toxicity and virtually no hemolytic activity. Here, liposomes containing the negatively charged phospholipid dioleoylphosphatidylserine (30%) showed some anti-inflammatory activity as well, although again these liposomes were less potent than those composed with DOPG [16]. Mixed micelles containing DOPG or DOPS (30% DOPG or DOPS, 20% soybean-derived phosphatidylcholine, and 50% sodium cholate, a bile salt) also inhibited TNFα production in peritoneal macrophages, although DOPG in this form was less potent than in liposomes [17].

Voelker and colleagues also provided some insight into the potential mechanism by which PG exerts its anti-inflammatory effects. It is known that the activation of TLR4 requires an accessory protein, MD-2, and a co-receptor, cluster of differentiation-14 (CD14). Less well known is the fact that TLR2 also requires CD14 [90,91,92]. Indeed, we recently showed that a CD14-blocking antibody inhibited the activation of both TLR4 and TLR2 in response to PAMPs and DAMPs [93]. Kuronama et al. [12] found that both CD14 and MD-2 can bind PG, and they have suggested that this binding interferes with the ability of these accessory proteins to bind microbial components such as LPS, thus inhibiting their capacity to promote TLR activation [94]. These investigators further showed that the potency of PG analogs to inhibit TLR4 correlates with their binding to CD14 [10], suggesting that this co-receptor might be the relevant target. In addition, as indicated previously, PG is not globally immunosuppressive, as it actually protects against infection and/or lung damage caused by multiple viruses, including respiratory syncytial virus and influenza A [95,96], and in fact, demonstrates some anti-viral activity [94]. In summary, the properties of PG, as well as its pro-proliferative, pro-differentiative, and anti-inflammatory effects in the absence of global immunosuppression, should be beneficial in promoting skin wound healing.

### 5.2. PG and Mitochondrial Function

Diabetes clearly impacts metabolism, although its effects on mitochondrial function are less clear. Nevertheless, in skin fibroblasts, mitochondrial function is impaired after treatment with AGEs [97], which, as mentioned, are formed from non-enzymatic glycation of proteins during hyperglycemia in diabetes [29] under conditions of increased oxidative stress. Similarly, methoxyglyoxal-induced AGEs elicit a decrease in mitochondrial membrane potential in these cells [98]; these results suggest that mitochondrial function is, in fact, impaired in diabetes. Mitochondrial function is also impaired in corneal endothelial cells from individuals with diabetes, with a resulting reduction in mitochondrial function and abnormal mitochondrial morphology [99]. Similarly, in monocytes/macrophages from subjects with gestational diabetes, decreased mitochondrial membrane potential and an enhanced production of ROS and inflammatory mediator were observed [100]. Finally, subtle alterations in cardiac mitochondria suggestive of dysfunction have been observed early in diabetes in a fructose-fed mouse model [101].

The involvement of PG in regulating mitochondrial function is not entirely clear. However, in mammalian cells, this phospholipid is a precursor of cardiolipin (essentially diphosphatidylglycerol [102]), which is known to be a key lipid in mitochondria. For the generation of cardiolipin, PG is first synthesized from phosphatidic acid, produced by the coupling of glycerol 3-phosphate and activated fatty acids. This phosphatidic acid then reacts with CTP to generate diacylglycerol-CDP. The activated diglyceride moiety in this precursor is transferred by phosphatidylglycerol phosphate synthase-1 to a second glycerol 3-phosphate to produce phosphatidylglycerophosphate, which is dephosphorylated to PG [103]. In eukaryotic cells, cardiolipin is formed by the reaction of this PG with the activated phosphatidyl group of another diacylglycerol-CDP. This final step is catalyzed by the enzyme cardiolipin synthase. In mammalian cells, cardiolipin is further remodeled by a process involving an enzyme called tafazzin, such that cardiolipins in mammals contain a high degree of unsaturation. Mutations in tafazzin lead to more saturated cardiolipin and the disease Barth syndrome, characterized by abnormal mitochondrial function, dilated cardiomyopathy, weakened skeletal muscles, and increased risk of infection due to neutropenia [104]. Despite some knowledge about cardiolipin synthesis, our understanding of the pathways involved in the normal metabolism of this lipid remains incomplete [105].

Nevertheless, cardiolipin is known to be critical for proper mitochondrial membrane structure and normal mitochondrial functioning, i.e., bioenergetics. Cardiolipin interacts with all of the electron transport chain complexes and is necessary for the integrity of their structure and their proper activity [106]. It is also important in the assembly of the F_1_-F_0_ ATP synthase, control of proton leakage, and maintenance of translocase complexes in the inner and outer mitochondrial membrane [105]. In addition, cardiolipin interacts with members of the Bcl2 family [105], and peroxidation of cardiolipin can lead to cytochrome C-mediated apoptotic cell death [106]. Finally, similar to PG, cardiolipin has also been reported to inhibit LPS-induced TLR4 activation, although this effect depends on the saturation state of the fatty acids. Thus, cardiolipin with a high degree of unsaturation, as would be found in the cardiolipin in mammalian mitochondria, inhibits LPS-induced TLR4 activation, whereas saturated cardiolipin, more typically found in microorganisms, actually stimulates TLR4 activity [107].

In addition to serving as a cardiolipin precursor, there is evidence to suggest that PG itself also has beneficial effects in mitochondria. This is perhaps not surprising considering the evolutionary link between mitochondria and prokaryotes, and the fact that, in bacteria, PG is important for protein folding and protein binding [108]. For example, PG promotes the assembly of chlorophyll-protein complexes in thylakoid membranes [109], and its deficiency reduces photosystem II assembly and activity [110] in cyanobacteria. These results are also consistent with findings in plants showing that PG can promote the assembly of photosynthetic electron transport complexes and is necessary for photosystem assembly in spinach [111]. Indeed, from the analyzed crystal structures, PG, in addition to cardiolipin, also seems to be an integral component of complexes III and IV in eukaryotic cells [102]. Furthermore, data in retinal pigment epithelium cells indicate that PG can protect against mitochondrial dysfunction [112], and both PG and cardiolipin restore the mitochondrial membrane potential in depleted mitochondria from the brain [113]. Additionally, and also supporting the effect of diabetes on mitochondrial function is a study regarding cardiac muscle in an STZ-induced diabetic mouse model in which ATP levels are reduced. Likewise, PG levels are decreased [114], with the mitochondria in these diabetic cardiomyocytes exhibiting greater cristae fusion [114]. In addition, in the cardiac tissue of diabetic rats, PG levels are reduced compared to normal rats or diabetic rats treated with insulin to prevent hyperglycemia [115], providing a potential link between decreased PG and impaired mitochondrial function in diabetes. Finally, Chen et al. [19] examined the effect of Kdo2-Lipid A, an almost homogeneous LPS substructure that acts as a specific activator of TLR4, on mitochondrial function in RAW 264.7 macrophage cells. These authors showed that Kdo2-Lipid A-induced TLR4 activation results in mitochondrial dysfunction. Importantly, supplementation of these cells with DOPG restores this reduced mitochondrial function [19]. This supplementation also reduced the expression of various pro-inflammatory genes, also suggesting a link between mitochondrial dysfunction and inflammation.

### 5.3. Other Possible Targets of PG

In addition to inhibiting TLR activation and promoting mitochondrial function, PG, and in particular DOPG, may target other effector enzymes as well. For example, in leukemia cells, PG has been shown to activate protein kinase C-betaII (PKCβII) [116], a splice variant that differs from PKCβI in its C-terminal region, and in fact, it is an amino acid sequence (FVNSEFLKPEVKS) in this region that has been shown to bind PG [117]. Another PKC isoform, PKCθ, has also been reported to be activated by PG, so it is possible that one or more PKC isoenzymes, as well as the so-called protein kinase-P (PK-P) identified by Klemm and Elias [118,119,120], may contribute to the observed effects of DOPG.

Another family of proteins that binds anionic phospholipids such as PG are the heat shock proteins (HSP). Some of these HSPs show affinity for PG, whereas others bind anionic phospholipids such as phosphatidylserine or phosphatidic acid. For example, HSP27, also known as HSPB1, binds phosphatidic acid but not PG, phosphatidylinositol, phosphatidylcholine, or phosphatidylethanolamine; interestingly, saturated fatty acid- and/or monounsaturated fatty acid-containing phosphatidic acid species bind more effectively than polyunsaturated fatty acid-possessing phosphatidic acids [105]. HSPA5, on the other hand, binds phosphatidylserine, in particular 1-palmitoyl-2-oleoylphosphatidylserine and cardiolipin, although neither PG nor phosphatidic acid was tested in this study [121]. HSP70 and mitochondrial HSP70 (HSPA1A and HSPA9, respectively) also bind 1-palmitoyl-2-oleoylphosphatidylserine and cardiolipin, but again PG and phosphatidic acid were not examined [122]. However, McCallister et al. [101] demonstrated that HSPA1A does, in fact, bind dipalmitoylphosphatidylglycerol and dipalmitoylphosphatidic acid, whereas another group found that HSPA1A only interacts weakly with PG [122]. These findings suggest that HSP family members may also be targets of DOPG.

### 5.4. Phospholipids, and in Particular PG, as Drug Delivery Systems

Lipids can be used to generate liposomes, such as the DOPG liposomes that, as we have shown, accelerate corneal epithelial wound healing in vitro and in vivo [84]; these liposomes are often used as drug delivery vehicles [123] since they are known to be taken up by cells (reviewed in [124]). DOPG, as well as other species of PG, can also be used in other types of lipid-based nanocarriers for drug delivery, including solid lipid nanoparticles, nanostructured lipid carriers, transfersomes, niosomes, nanoemulsions, ethosomes, and lipidoid nanoparticles, as extensively reviewed by Kandregula et al. [125] and Kumari et al. [126]. In turn, these lipid-based nanocarriers are being investigated as systems to deliver proteins, nucleic acids, and small molecule drugs to diabetic ulcers to stimulate wound healing [125]. However, in this case, the phospholipids from which the nanocarriers are generated are considered inactive ingredients, whereas, based on our and others’ data, we propose that PG, and in particular DOPG, can, in fact, actively contribute to diabetic wound healing. Nevertheless, it is possible that there may be a better mechanism for delivering DOPG to diabetic wounds than liposomes, such that the phospholipid is more efficacious in its ability to inhibit inflammation, improve mitochondrial function, and promote wound healing.

### 5.5. Dioloeoylphosphatidylglycerol to Treat Chronic Diabetic Wounds

Diabetic and pressure ulcers of the skin are an important determinant of morbidity and mortality in the diabetic and aged population [127], as well as in those with spinal cord injuries [128], especially as these wounds often become chronic and fail to completely heal. We have previously shown that egg PG accelerates skin wound healing [82] and that DOPG enhances corneal epithelial wound healing [84]; these data indicate the ability of PG to promote epithelial healing. DOPG also inhibits inflammation, a hallmark of chronic wounds in patients with diabetes, a disease known to be associated with a chronic inflammatory state [129]. In addition, serum levels of the high mobility group, box 1 (HMGB1) protein are elevated in patients with diabetes [130]. HMGB1 is reportedly a DAMP [131], functioning to activate pattern recognition receptors such as TLR4. In a recent study, we showed that HMGB1 activated TLR4, as monitored using a reporter cell line; importantly, DOPG inhibited TLR4 activation induced by HMGB1 [93]. In addition, several TLRs, including TLR2 and TLR4, are up-regulated in diabetic wounds, and TLR2- and TLR4-deficient knockout mice show accelerated wound healing [46]. The antimicrobial peptide S100A9 is increased in chronic wounds [46], and we have shown that S100A9 activates both TLR2 and TLR4; this S100A9-induced activation is inhibited by DOPG [15,93]. Thus, as noted above, the pro-proliferative, pro-differentiative, and anti-inflammatory actions of DOPG should be beneficial in promoting the healing of chronic skin wounds.

**Figure 6:**
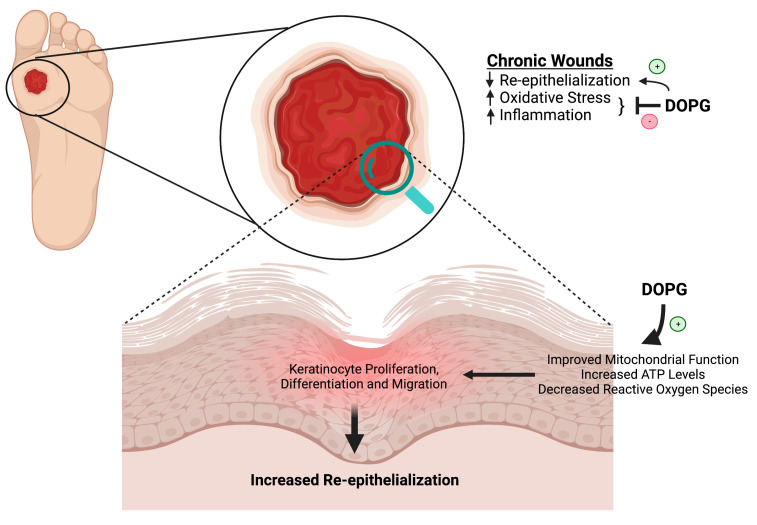
Chronic wounds are characterized by poor re-epithelialization, increased oxidative stress, and inflammation. As discussed in this review, data in the literature show that DOPG improves mitochondrial function, which suggests that DOPG might enhance wound healing via this mechanism to increase ATP levels and decrease reactive oxygen species. We have shown that DOPG also promotes keratinocyte proliferation, which should help to restore epidermal integrity. In addition, DOPG’s anti-inflammatory effects should also result in decreased oxidative stress and increased healing even of chronic wounds, such as can occur with diabetes. Created with Biorender.com.

In addition, DOPG’s ability to enhance mitochondrial function should lead to reduced oxidative stress (less superoxide production because of better mitochondrial efficiency) and the increased generation of ATP; this ATP can then provide the “power” to enhance keratinocyte migration, proliferation, and differentiation to complete the wound healing process. Finally, PG is a naturally occurring phospholipid that is already present in a number of human therapies—often listed as an inactive ingredient. For example, PG is a constituent of more than a dozen skincare products [132], eye drops (Systane Complete Lubricant eye drops), and other medications (e.g., Visudyne, which is a Food and Drug Administration (FDA)-approved injectable photoenhancer for photodynamic treatment of age-related macular degeneration [133] and in multiple surfactant therapies used to treat acute respiratory distress syndrome in infants [134]). Therefore, DOPG liposomes may be a novel, safe, and effective treatment to enhance the healing of chronic skin wounds and individuals with diabetes, as illustrated in Figure 6. However, there are other factors that are known to contribute to the delayed healing of diabetic wounds, and it is unclear as to whether DOPG would affect these elements or not. For example, neuropathy and hypoxia as a result of vasculopathy are also involved in the pathogenesis of chronic diabetic wounds [125]; the effect of DOPG on these other aspects contributing to impaired diabetic wound healing is as yet unknown.

## 6. Conclusions

In this review, we proposed the use of DOPG liposomes as a treatment to improve the healing of diabetic skin wounds based on their anti-inflammatory properties and their ability to enhance mitochondrial function. However, as mentioned in Section 5.4, liposomes may not be the ideal application method to ensure the efficacy of DOPG in terms of these desired effects. The key advantage of liposomes, as pointed out above, is the fact that, because they are already in use in several FDA-approved medications, their safety is assured; additional examples of liposomal delivery systems already in use in drugs with FDA approval are discussed in reference [135]. However, there are now a number of non-liposomal lipid-based nanoparticle drug delivery systems that are in clinical trials, although the majority by far are still liposomal [135]. If these drug delivery approaches demonstrate appropriate efficacy and safety, these trials may pave the way for improved techniques to administer DOPG. Even larger numbers of lipid-based drug delivery systems are being investigated in pre-clinical models, with many demonstrating efficacy, including for enhancing diabetic wound healing [125]. In these studies the lipid-based nanoparticles are used to deliver proteins (such as epidermal growth factor), nucleic acids (e.g., small interfering RNAs or microRNAs), and small-molecule drugs (such as antibiotics). We speculate that using lipid-based nanoparticles composed of DOPG would increase the efficacy of the delivered agents in terms of their ability to stimulate diabetic wound healing since both the cargo and the lipid “packaging” would exert beneficial effects. As the treatment options currently available to treat diabetic wounds are suboptimal and associated with challenges [125], studies investigating the utility of DOPG to treat diabetic skin wounds, as proposed in this review, could help to show DOPG’s value in promoting the healing of chronic diabetic wounds and allow for the fulfillment of its potential as a therapy for this significant clinical problem.

## Figures and Tables

**Table 1:** Fatty Acid Composition of Egg-derived PG ^1^.

Fatty Acid	Percentage
Palmitic acid (16:0) ^2^	32.9
Palmitoleic acid (16:1)	0.9
Stearic acid (18:0)	12.2
Oleic acid (18:1)	30.2
Linoleic acid (18:2)	18.7
Arachidonic acid (20:4)	3.5
Docosatetraenoic acid (22:4)	0.9
Docosapentaenoic acid (22:5)	0.7

^1^ Egg PG from Avanti Polar Lipids, Inc. (Alabaster, AL); some lot-to-lot variability is possible. ^2^ The first number in parentheses represents the number of carbon atoms in the fatty acid, and the second number (after the colon) represents the number of double bonds.

**Table 2:** Effects of PG Species on Keratinocyte Proliferation *.

PG Species	Number of Carbons: Number of Double Bonds in R1; R2 Fatty Acids	Effect on Keratinocyte Proliferation (at Concentrations from 6.25–100 µg/mL)
egg-derived phosphatidylglycerol	see Table 1	↑ Slowly proliferating cells ↓ Rapidly proliferating cells
dipalmitoylphosphatidylglycerol	16:0; 16:0	ns
dioleoylphosphatidylglycerol	18:1; 18:1	↑
1-palmitoyl-2-oleoyl-phosphatidylglycerol	16:0; 18:1	↑
distearoylphosphatidylglycerol	18:0; 18:0	↑
1-palmitoyl-2-linoleoyl-phosphatidylglcerol	16:0; 18:2	↓
dilinoleoylphosphatidylglycerol	18:2; 18:2	↓
1-palmitoyl-2-arachidonoyl-phosphatidylglycerol	16:0; 20:4	↓
dihexanoylphosphatidylglycerol	6:0; 6:0	ns
soy-derived phosphatidylglycerol	17% 16:0, 6% 18:0, 13% 18:1, 59% 18:2 and 5% 18:3	↓

* Results from reference [83]. ns = not significant.

## Data Availability

No new data were created or analyzed in this study. Data sharing is not applicable to this article.
